# Aplasia Cutis Congenita of the Scalp with a Familial Pattern: A Case Report

**Published:** 2016-09

**Authors:** Waleed AlShehri, Sara AlFadil, Alhanouf AlOthri, Abdulaziz O. Alabdulkarim, Shabeer A. Wani, Sari M. Rabah

**Affiliations:** Department of Plastic and Reconstructive Surgery, King Fahd Medical City, Riyadh, Saudi Arabia

**Keywords:** Aplasia cutis congenital, Scalp, Reconstruction, Familial

## Abstract

Aplasia Cutis Conginita (ACC) is a condition characterized by congenital absence of skin, usually on the scalp. ACC can occur as an isolated condition or in the presence of other congenital anomalies. Here we describe a case of a 16 days old baby girl with an isolated ACC of the scalp. Her elder two siblings have been diagnosed with ACC with concomitant cardiac or limb anomalies. The patient was managed conservatively until the defect has scarred 6 months later.

## INTRODUCTION

Cutis aplasia or Aplasia Cutis Congenita (ACC) is an uncommon and rare congenital abnormality involving variant layers of the skin, mostly as a solitary lesions involving the midline over the skull vertex; and less commonly, underlying periosteum and bone.^1^ Other sites may occur as well on the chest, abdomen or limbs.^2^^,^^3^ Of lesions on the scalp, 20% can involve the cranium, exposing the underlying dura membrane. ACC could also be found in other congenital anomalies; since it was first described in 1767 by Cordon, around 500 similar cases have been reported so far.^4^ Different anomalies were classified into 9 groups based on the number and the presence or absence of other anomalies (Table 1).^5^ The lesions in those cases are quite variable, ranging from only local absence of skin to a complete absence of epidermis, subcutaneous tissue, bone, or in some cases the dura.^6^^-^^8^ The incidence of ACC is estimated as 1 per 10,000 live births.^1^

**Table 1:** Classification for ACC

**Group**		**Associated Anomalies**	**Inheritance**
1	Scalp ACC without multiple anomalies	Cleft lip and palate, tracheoesophageal fistula, patent ductus arteriosus, omphalocele, mental retardation, polycystic kidneys	Autosomal dominant or sporadic
2	Scalp ACC with limb abnormalities	Limbs reduced, syndactyly, clubfoot, encephalocele, nail dystrophy or absence, persistent cutis marmorata	Autosomal dominant
3	Scalp ACC with skin/ organoid nevi	Epidermal nevi, organoid nevi, corneal opacities, scleral dermoids, eyelid colobomas, mental retardation, seizures	Sporadic
4	ACC overlying embryologic malformations	Meningomyeloceles, spinal dysraphia, cranial stenosis, leptomeningeal angiomatosis, gastroschisis, congenital midline porencephaly, ectopia of ear, omphalocele	Depends upon underlying condition
5	ACC with fetus papyraceus or placental infarcts	Single umbilical artery spastic developmental delay, spastic paralysis, clubbed hands and feet, amniotic bands	Sporadic
6	ACC associated with epidermolysis bullosa	Blistering of skin and/or mucous membranes, deformed nails, pyloric or duodenal atresia, abnormal ears and nose, ureteral stenosis, renal anomalies, amniotic bands	Depends upon type of epidermolysis Bullosa
7	ACC localized to extremities without blistering	None	Autosomal Dominant or Recessive
8	ACC caused by teratogens	Imperforate anus (methimazole), other signs of intrauterine infection with varicella or herpes simplex	Not Inherited
9	ACC associated with congenital syndromes	Trisomy 13, 4p-syndrome, ectodermal dysplasias, ocal dermal hypoplasia, amniotic band disruption omplex, XY gonadal dysgenesis, Johanson-Blizzard syndrome	Depends upon syndrome

This failure of formation is frequently more observed in females. The etiology remains unclear so far; however, both genetic and environmental causes have been implicated, including vascular blood supply, a sudden arrest of midline embryological development, failure in neural tube closure, and syphilis have at one time contributed as the cause.^1^^,^^9^ Rupture of amniotic membrane in an early time, forming amniotic bands, may also be from the cause.^5^ A number of teratogenic drugs such as methimazole, is a thiomedazole derivative used as an anti-thyroid agent, have shown to be involved.^10^^-^^13^

There are similar cases, classified as being of an autosomal dominant inheritance.^14^ Establishing a diagnosis is usually based on the findings of the clinical examination, typically presenting as a hairless, smooth skin defect covered up by atrophic tissue or a dark-colored eschar. Superficial defects presenting as an ulcer are usually treated conservatively. Extensive or deep defects may require reconstruction of the scalp area or the use of bone transplants. However, hairless or scarcely haired scars mandate excision of the lesion and covering it with local flap from the scalp.^9^^,^^15^^-^^20^

## CASE REPORT

A 16 days old newborn female from Saudi Arabia was presented to the clinic with a skin defect localized on the scalp since birth. The baby did not suffer from any ailments, and her medical history was unremarkable. Her mother, 32 years old, denied any history of illnesses during her pregnancy, infection or drug intake taking including Non-Steroidal Anti-Inflammatory Drugs (NSAID) or methimazole. She completed 38 weeks of gestation, and delivered her baby via a normal vaginal delivery. 

**Fig. 1:**
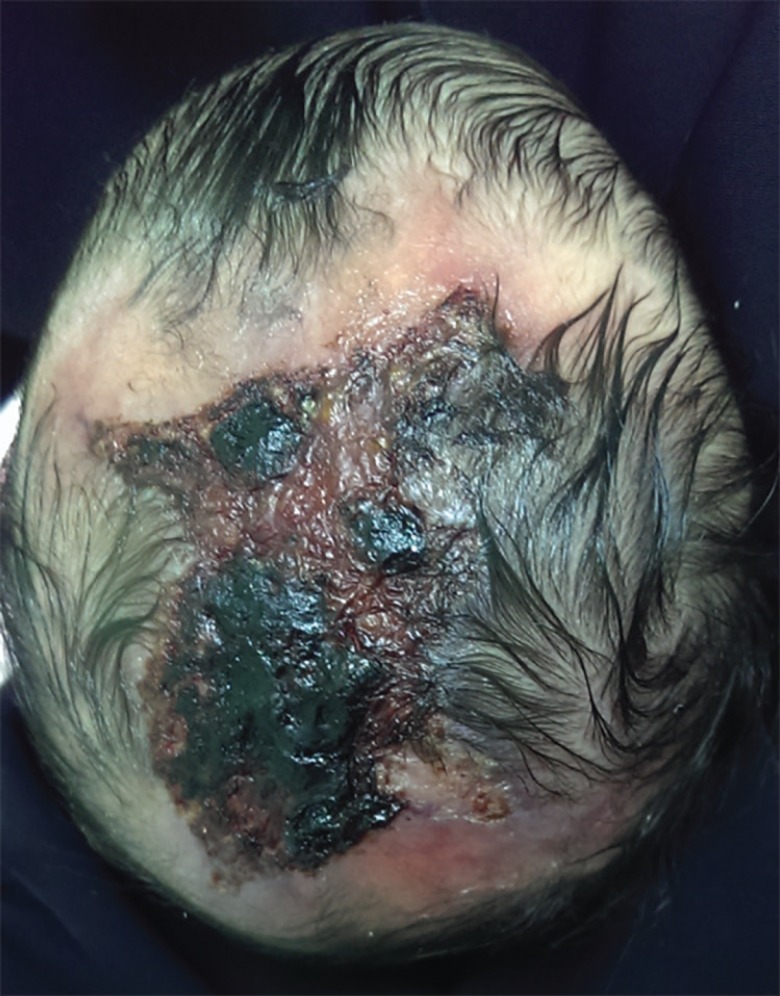
The newborn presented with skin defect of the scalp with an overlying crust

The newborn did not sustain any birth injury and did not suffer from any other abnormalities or feeding difficulties. She did not require any intensive care, and went home from hospital with her mother. Upon local examination, the defect was solitary, localized with an irregular shape and approximately 6×6 cm in size (Figure 1). The lesion involved the epidermis and the upper dermis only. Neurosurgical team was involved in the care of this patient. A CT Scan of the head was performed, and no deep tissue involvement was noted.

**Fig. 2:**
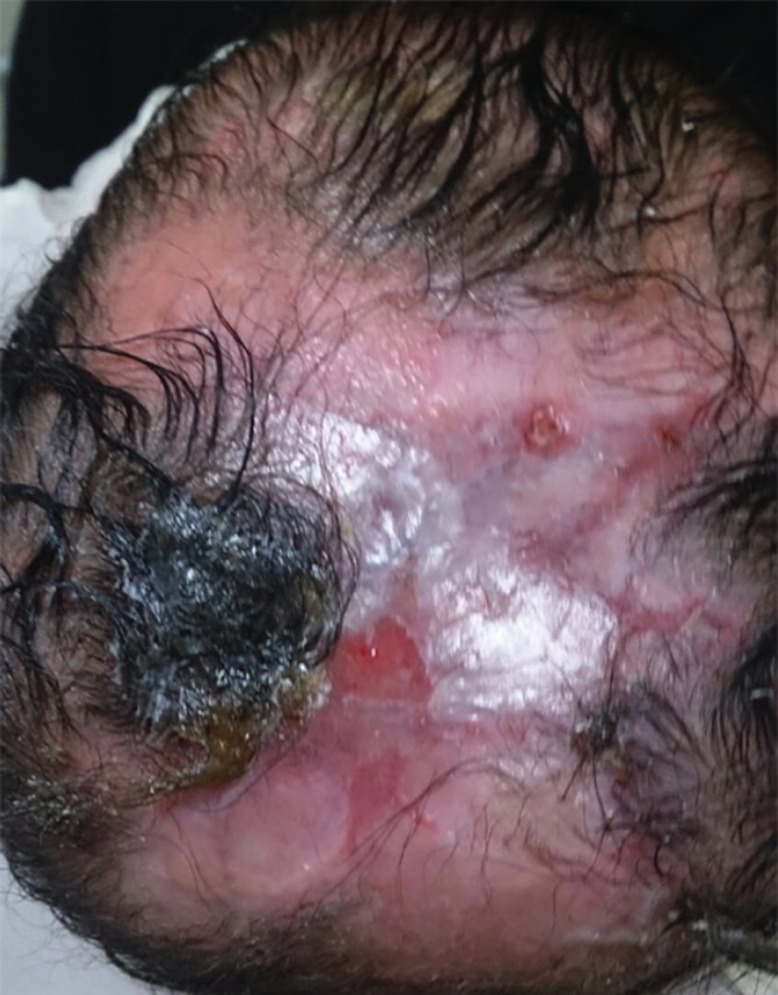
Six months follow up shoes scar formation over the affected area

**Fig. 3:**
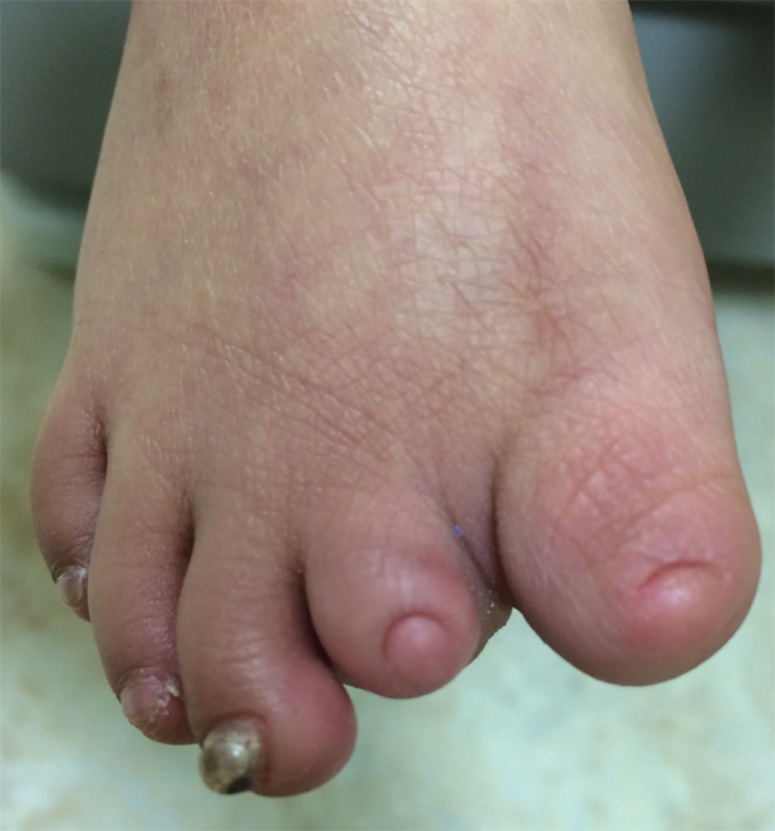
Unilateral terminal reduction of the right first and second tow

Reconstruction solutions were offered to the parents but they insisted on non-surgical intervention. Therefore, the patient was treated with non-invasive debridement of the lesion and local therapy, including gentle water cleansing and the application of topical antibiotic ointment. 6 months later, the patient has returned for a follow up. Scar tissue has formed over the defect (Figure 2). Family history revealed that none of her parents had the same condition; however, two of her sisters did, and were diagnosed with cutis aplasia. The elder one is currently 4 years of age, with right unilateral terminal reduction of the first and second toes (Figure 3). The other sister was born prematurely and died shortly after birth due to cardiac anomalies.

## DISCUSSION

ACC occurs as a solitary defect, it can happen alone or in the presence of syndromic congenital anomalies. The involvement of the scalp area may lead to the understanding of the etiology. Upon our review to the literature available, cases were often characterized by an entire absence of skin and subcutaneous tissues. Histologically, we found that most of the lacking tissues belonged to epithelial ectoderm. The condition could be associated with Chromosomal defects.^21^ Some researches showed the association with gestational conditions such as an intrauterine vascular ischemia, amniotic adherences, and viral infections.^22^^,^^23^

A rise of alpha-fetoprotein levels and a distinct amniotic fluid acetylcholine sterase band were found in recent article as markers for ACC.^24^ Also a number of drugs have been linked to ACC. For example, the use of cocaine during pregnancy can lead to vasoconstriction of the placenta or disruption of the fetus vascularity, causing the cranial defects and anomalies of the central nervous system (CNS).^25^ Methimazole, a drug used for the treatment of hyperthyroidism, may show some skin affection.

Benzodiazepines use is also linked with ACC*.*^7^ Surgical treatment requires careful preoperative planning.^26^ Minimal superficial lesions are treated conservatively to heal gradually by re-epithelialization and result with a hypertrophic or atrophic scar. Tissue expander insertion may be necessary in extensive lesions reaching the scalp; whereas the one deep enough to reach brain, bone and meningeal transplants may be indicated.^9^^,^^15^^,^^20^^,^^27^ Deep defects overlying the sagittal sinus are indicators for urgent surgical intervention to prevent potentially lethal infections or hemorrhage.^28^^-^^30^

Grafting,^31^ biological dressings use temporarily,^32^ and silver sulfadiazine dressings while waiting for the processes of skin and bony ingrowth have been published with variable degree of success.^33^

## CONFLICT OF INTEREST

The authors declare no conflict of interest.
